# Review of Molecular Tools Used in Diagnosis of *Babesia* spp. and *Anaplasma* spp. Infection in Wild Boar and Their Ticks—20 Years Retrospective Review

**DOI:** 10.3390/ani15152211

**Published:** 2025-07-27

**Authors:** Ioan Cristian Dreghiciu, Diana Hoffman, Mirela Imre, Ion Oprescu, Simona Dumitru, Tiana Florea, Sorin Morariu, Vlad Iorgoni, Anamaria Plesko, Gabriel Orghici, Marius Stelian Ilie

**Affiliations:** 1 Department of Parasitology and Parasitic Disease, Faculty of Veterinary Medicine, University of Life Sciences “King Mihai I” from Timisoara, 119 Calea Aradului, 300645 Timisoara, Romania; diana.hoffman@usvt.ro (D.H.); ionoprescu@usvt.ro (I.O.); tijana.florea@usvt.ro (T.F.); sorinmorariu@usvt.ro (S.M.); anamaria.plesko@usvt.ro (A.P.); mariusilie@usvt.ro (M.S.I.); 2 Veterinary and Food Safety Directorate 4, Surorile Martir Caceu, 300585 Timisoara, Romania; simonagiubega@gmail.com; 3 Department of Infectious Diseases and Preventive Medicine, Faculty of Veterinary Medicine, University of Life Sciences “King Mihai I” from Timisoara, 119 Calea Aradului, 300645 Timisoara, Romania; vlad.iorgoni@usvt.ro (V.I.); gabriel.orghici@usvt.ro (G.O.)

**Keywords:** *Babesia* spp., *Anaplasma* spp., PCR, ELISA, wild boar, ticks, diagnostic methods

## Abstract

Wild boars (*Sus scrofa*) and their associated ticks represent important reservoirs and vectors for a variety of zoonotic pathogens, particularly *Anaplasma* spp. and *Babesia* spp. The aim of this review is to highlight the diagnostic methods employed throughout the past two decades to detect these pathogens in wild boars and their associated ticks, focusing especially on PCR methods (conventional and real-time), on sample origin (host or vector), and on specific primers. This review compiles data from studies conducted between 2005 and 2024, providing a comparative overview of targeted genes, such as groEL, msp2, and 16S rRNA for *Anaplasma*, and 18S rRNA for *Babesia*. Despite the prevalence of molecular diagnostic techniques, serological methods like ELISA have not been applied to wild boar or tick samples in this context. The review also analyzes geographical trends, with a higher research output noted in Central and Eastern Europe, Japan, and the United States. The performance of the methods is discussed based on reproducibility, sensitivity, specificity, pathogen prevalence, and technical variables. By synthesizing these findings, the review identifies methodological gaps and offers a reference framework for improving diagnostic accuracy in wildlife disease surveillance.

## Simple Summary

Zoonotic pathogens like *Anaplasma* and *Babesia* species are frequently present in wild boars and their associated ticks. Monitoring and managing tick-borne illnesses that can impact both humans and animals requires an understanding of the distribution of these infections in wildlife. Findings from the past 20 years have been compiled in this study, highlighting the most frequently employed genetic targets, molecular techniques, especially different types of Polymerase Chain Reaction (PCR), and biological material used for testing. The review outlines the main diagnostic techniques used to identify *Babesia* and *Anaplasma* in wild boars and their associated tick vectors. Additionally, it identifies research gaps, providing assistance for future initiatives to enhance disease surveillance and diagnostic reliability in animals.

## 1. Introduction

The analysis of biological samples provides a comprehensive approach for detecting these pathogens. These arthropods survive in a variety of environments, including alpine meadows, lakes, woods, and semi-arid regions. They have become more prevalent in urban and suburban areas in recent years, as seen in several European cities [1].

The occurrence of infectious diseases in wild boars is closely linked to the presence of appropriate vectors. Wild boars can serve as asymptomatic carriers of *Babesia* spp. and *Anaplasma* spp., harboring these pathogens without developing clinical disease. Notably, the spatial distribution of host species does not necessarily affect the prevalence of vector species within a given region or dictate infection rates [2]. Research investigating the correlation between host presence and tick species diversity suggests that tick populations are not solely dependent on the variety of available hosts.

Ticks serve as major vectors for a range of parasitic and infectious diseases that affect both domestic pigs and wild boars. In Europe, the most notable tick-borne diseases impacting swine include monocytic ehrlichiosis, African Swine Fever (ASF), babesiosis, and Lyme borreliosis [3]. The abundance and geographic range of tick populations are influenced by environmental factors such as climate and vegetation. Seasonal variations also play a crucial role, with adult ticks demonstrating an increased capacity for disease transmission during humid periods, whereas the larval and nymphal stages are more prevalent in drier conditions [4].

A wide range of diagnostic methods are utilized to detect tick-borne infections; however, the accuracy of these techniques depends on multiple factors, including the stage of disease progression, parasite load, diagnostic sensitivity and specificity, and the experience of the examining physician. For instance, in chronically infected animals, piroplasms persist in the bloodstream at levels sufficient to sustain tick infection but often fall below the detection threshold of standard diagnostic tests [5].

Diagnostic tools may be employed individually or in conjunction with other criteria to enhance the accuracy of disease identification. To be deemed reliable for veterinary or medical use, diagnostic assays must meet specific validation criteria [6,7].

The performance of these methods is evaluated based on standardized benchmarks, providing critical data on reproducibility, sensitivity, specificity, pathogen prevalence, genetic variability, and methodological factors such as antigen-antibody interactions, manual versus automated processing, and overall test standardization. These variables collectively determine the efficacy and diagnostic reliability of the methods employed in pathogen detection [7].

The aim of this review is to provide a comprehensive overview of the molecular diagnostic methods used over the past two decades for the detection of *Babesia* spp. and *Anaplasma phagocytophilum* in wild boars and their associated ticks, with a particular focus on the type of samples analyzed, PCR approaches applied, and the specific primers employed in each study.

## 2. Materials and Methods

A comprehensive study was used as a starting point in order to determine the diagnostic methods cited in literature for the detection of *Anaplasma* and *Babesia* species in wild boars as well as their tick vectors. Google Scholar, Scopus, and PubMed were the three main databases used in the review. The timeline for the selected studies was 2005–2024. The keywords used in various combinations were “wild boar,” “ticks”, “ELISA,” “PCR,” “*Babesia*,” and “*Anaplasma*”.

M.I., D.H., and A.P. were responsible for identifying and selecting studies from PubMed, Scopus, and Google Scholar, following established inclusion criteria appropriate for a systematic review.

A total of 910 articles were retrieved by the searches: 876 from Google Scholar (29 deemed appropriate after filtering), 18 from PubMed (17 maintained), and 16 from Scopus (11 relevant). Articles that published primer sequences, detailed diagnostic techniques (e.g., PCR, ELISA), had appropriate language (English) clarity, were the accepted publication type (peer-reviewed articles), referred to host species (wild boar and associated ticks), had a clear focus on molecular diagnostic tools and offered pertinent information on the identification of these infections in ticks or wild boars were included. Studies that did not focus on diagnostic, overlapped with other studies, or lacked methodological clarity were excluded.

Twenty-three pertinent studies made up the final selection. Sample types, PCR procedures, primer sequences, and molecular or serological methods were all covered using a qualitative content analysis methodology. This made it possible to compare the diagnostic approaches used in various research designs and geographical areas. The overview is organized according to the disease, emphasizing the molecular targets of the diagnostic instruments used, as well as the animal or vector source of DNA or antigens.

This review was conducted in accordance with the PRISMA (Preferred Reporting Items for Systematic Reviews and Meta-Analyses) guidelines to ensure methodological transparency and reproducibility throughout the search, selection, and data synthesis processes.

No meta-analysis was conducted due to substantial methodological heterogeneity among the included studies, which varied in their diagnostic targets, sample types, study populations, and outcome reporting. As a result, a structured qualitative synthesis was performed instead to identify consistent patterns, research gaps, and emerging trends in molecular diagnostics for *Babesia* and *Anaplasma*. Given this heterogeneity and the lack of uniform study designs or comparable effect measures, a formal risk of bias assessment was not considered appropriate.

We considered potential sources of bias narratively to help interpret the evidence from the included studies, without formally scoring their risk of bias.

## 3. Results

**Figure 1:**
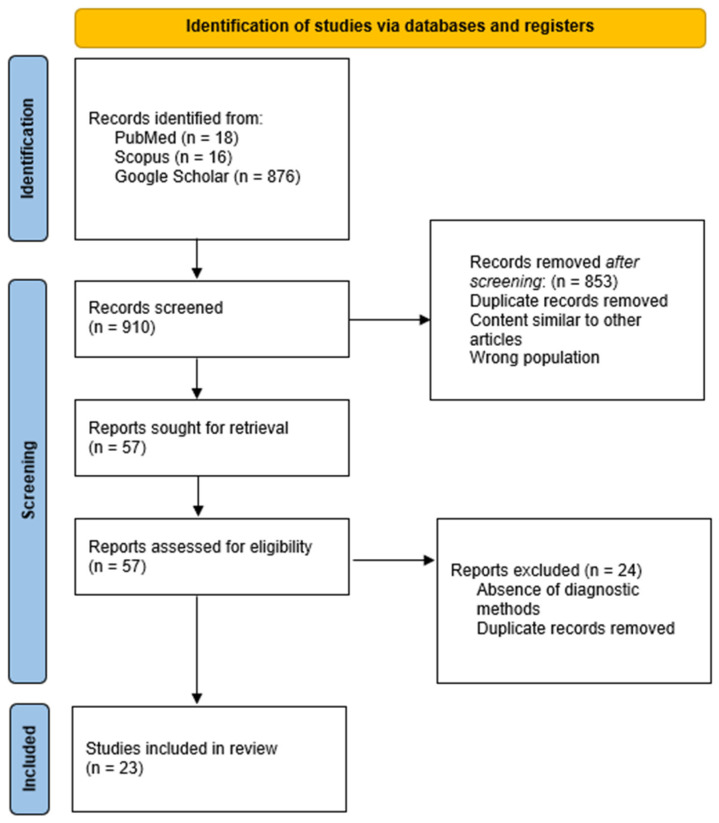
Flowchart of study selection [8].

In accordance with the PRISMA (Preferred Reporting Items for Systematic Reviews and Meta-Analyses) guidelines, we conducted a structured literature screening and selection process. A total of 910 records were initially identified across three databases (PubMed, Scopus, and Google Scholar). After removing duplicates, 57 records remained for screening. Following full-text assessment, 23 studies met the eligibility criteria and were included in the final review. The review selection process is summarized in Figure 1, which illustrates the flow of articles through the stages of identification, screening, eligibility assessment, and inclusion.

The initial search retrieved a high number of results, particularly from Google Scholar, which indexes a broad spectrum of sources, including dissertations, conference abstracts, and non-peer-reviewed documents. After removing duplicates, these records were screened based on strict inclusion criteria (language, host species, focus on molecular diagnostic tools, and publication type). As a result, many documents were excluded for not meeting the scientific and methodological standards of this review, which explains the substantial reduction from the initial records to the final count of included studies (Table 1).

## 4. Epidemiological and Clinical Diagnosis

### 4.1. Diagnosis of Babesia Infection

Babesiosis is a tick-borne disease caused by *Babesia* species, with vectors belonging to the *Dermacentor*, *Haemaphysalis*, *Hyalomma*, and *Rhipicephalus* genera. Clinically, the disease manifests with anemia, jaundice, and haemoglobinuria, with severe cases potentially leading to fatal outcomes [9].

In pigs and wild boars, babesiosis is a multifaceted disease influenced by various environmental and parasitic factors. Ongoing research aims to enhance the understanding of its etiology and epidemiology [10]. Notably, jaundice has not been previously documented in cases attributed to *Babesia trautmanni* as the causative agent [11,12]. Variations in clinical presentation and disease severity may be linked to differences in pathogen virulence or host immune responses. Zobba et al. (2014) proposed that distinct strains, detected in both clinically affected and asymptomatic pigs, could contribute to variations in pathogenicity [13].

The diagnosis of porcine babesiosis may be overlooked if peripheral blood smears are not conducted or if the disease is not considered within the differentials category, particularly in regions with a historically low prevalence of tick-borne diseases. Macroscopic findings such as jaundice, anemia, and haemoglobinuria lack specificity. Thus, differential diagnoses in pigs should include anaplasmosis, *Eperythrozoon suis* infection, and acute leptospirosis [11].

Among swine, *B. trautmanni* is the most frequently identified *Babesia* species, with *Rhipicephalus* spp. serving as its primary vector. In South Africa, all documented cases of porcine babesiosis were reported within pig production units during the 1940s and 1950s, with *B. trautmanni* identified based on morphological analysis of parasites in blood smears [12,14]. The natural reservoirs of *B. trautmanni* are believed to be warthogs (*Phacochoerus africanus*) and bush pigs (*Potamochoerus larvatus*). Furthermore, based on similarities in piroplasm size and genetic homology, *Babesia* sp. *Suis* is considered closely related to *B. trautmanni* [15,16,17].

The transmission of babesiosis between hosts is contingent upon both the suitability of the invertebrate vector and the competence of the vertebrate host in maintaining the pathogen in an infectious state [18]. Environmental factors, including temperature, humidity, and precipitation, play a significant role in regulating tick activity [19,20]. Increasing global temperatures may contribute to the expansion of tick habitats, thereby facilitating the spread of *Babesia* species into previously unaffected animal populations [21,22,23]. Additionally, rising human populations and habitat fragmentation have intensified interactions between reservoir hosts (such as wild boars and pigs for *Babesia trautmanni*) and domestic swine, creating conditions conducive to pathogen transmission. Direct exposure to infected carriers enhances the likelihood of transmission, as the duration of tick attachment directly correlates with the efficiency of *Babesia* spp. sporozoite inoculation [18].

The rapid and continuous intraerythrocytic replication of *Babesia* spp. leads to both intravascular and extravascular hemolysis, resulting in anemia due to the destruction of erythrocytes by emerging parasites [24,25,26]. Intravascular hemolysis is responsible for haemoglobinuria and, in severe cases, acute tubular necrosis [27,28,29,30]. Conversely, extravascular hemolysis contributes to bilirubinemia and hepatic cholestasis, which clinically manifest as jaundice. Additional clinical signs, including lethargy, lameness, and fever, arise because of diminished oxygen transport due to red blood cell depletion [15,18]. Compensatory mechanisms, such as extramedullary hematopoiesis (EMH) and regenerative anemia, are characterized by reticulocytosis and polychromasia in response to erythrocyte loss. Thrombocytopenia, a hallmark of babesiosis, is frequently observed and results in petechial hemorrhages, though the precise mechanisms underlying platelet dysfunction remain undetermined [31,32]. While acute lung injury and pneumonia are less common, they have been documented in canine infections caused by *Babesia rossi* as well as in human cases [32,33,34].

### 4.2. Diagnosis of Anaplasma Infection

A wide range of wild and domestic animals are susceptible to infection by *Anaplasma phagocytophilum*. However, clinical diseases have been reported in only a limited number of species, including domestic ruminants, horses, cats, dogs, and humans [35,36,37,38]. While dogs are capable of being infected with *A. phagocytophilum*, they are primarily considered incidental hosts, and their role as potential reservoirs remains uncertain [36,39]. As an obligate intracellular bacterium, *A. phagocytophilum* requires reservoir hosts that can sustain its survival, particularly during periods of reduced tick activity [40].

In wild boars, *A. phagocytophilum* has been identified at low prevalence in the Czech Republic [41] and Slovenia [42]. A more recent study in Poland reported a 12% prevalence of the bacterium in wild boars [43]. Notably, in both Slovenia and Poland, gene sequences of *A. phagocytophilum* detected in wild boars were identical to those found in human cases and in *Ixodes ricinus* ticks [42,44]. In Sicily, there is evidence suggesting that pigs may also be susceptible to *A. phagocytophilum* infection [44]. However, in central and southern Spain, where *I. ricinus* is relatively rare [45], *Anaplasma* spp. has not been documented in wild boars [46,47,48]. However, other tick species that parasitize wild boars in the region have tested positive for *A. phagocytophilum* DNA [46].

#### 4.2.1. Transmission and Clinical Manifestations

*Anaplasma phagocytophilum* is transmitted to hosts by ticks within 24 to 48 h after initiation of feeding [49,50]. However, in dogs, a successful establishment of the infection appears to require a minimal inoculation dose [50].

Bacteremia typically develops between four and seven days post-tick bite in naturally acquired infections, whereas experimental blood inoculation leads to bacteremia within three to four days. This suggests that during the early stages of infection, the bacterium either remains at undetectable levels in the bloodstream or replicates in alternative cellular environments. Cell surface analyses indicate that endothelial cells within the microvasculature provide an optimal site for *A. phagocytophilum* dissemination of peripheral blood granulocytes [51].

The clinical severity of anaplasmosis varies widely, ranging from mild subclinical presentations to severe acute disease [36,52]. Severe manifestations are often associated with co-infections, host immune responses, and variations in strain pathogenicity [53]. Anaplasmosis is a multisystemic, acute disease characterized by diverse clinical and pathological manifestations due to its potential impact on multiple organ systems. Following an incubation period of one to two weeks, the most observed clinical signs include fever, lethargy, inappetence or anorexia, weight loss, and musculoskeletal pain or discomfort [53,54]. Fever presents inconsistently, occurring in 46–100% of cases [55,56], with recorded temperatures ranging from 39.2 °C to 41.5 °C [56,57,58,59,60]. Typically, fever coincides with peak bacteremia and persists for less than a week [55].

Lymphadenopathy, splenomegaly, and hepatomegaly are frequently reported in anaplasmosis cases. Splenomegaly has been documented in 12–100% of naturally infected animals [56,61]. Studies in canine and murine models of *A. phagocytophilum* infection indicate that lymphadenopathy and splenomegaly result from reactive lymphoid hyperplasia, accompanied by extramedullary hematopoiesis in the spleen, enlargement of active lymph nodes, and an increased presence of macrophages and plasma cells within the red pulp [49,60,62].

#### 4.2.2. Other Clinical Signs

Additional clinical manifestations of *A. phagocytophilum* infection include gastrointestinal disturbances, polyuria, polydipsia, respiratory symptoms, pale mucous membranes, bleeding disorders, uveitis, scleral congestion, polymyositis, and neurological abnormalities [58,60,63]. Respiratory symptoms commonly observed in affected individuals include dyspnea, tachypnea, and coughing, although the cough is typically mild, infrequent, and non-productive [56].

However, two independent studies found no significant association between *A. phagocytophilum* infection and neurological manifestations [64,65].

### 4.3. Diagnosis of Tick Parasitism

Ticks are significant ectoparasitic arthropods that contribute to considerable economic losses due to both their direct and indirect effects on hosts. As obligatory blood feeders, they serve as vectors for a wide range of pathogens affecting both domestic and wild animals. Ticks rank second only to mosquitoes in their role as major vectors of zoonotic pathogens [21,66]. Globally, over 900 tick species have been identified, transmitting a diverse array of viral, bacterial, and protozoan pathogens to their hosts [67].

The role of domestic and companion animals in the transmission of tick-borne diseases has been widely investigated across various regions, including South Africa [68,69,70]. However, the extent to which wild animals function as sentinels for both animal and human diseases remains poorly understood. Additionally, there is limited data on the involvement of wild and domestic animals in South Africa as reservoirs for zoonotic pathogens such as *Rickettsia* spp., *Borrelia burgdorferi*, *Anaplasma phagocytophilum*, and *Coxiella burnetii* [69].

The primary indigenous tick species that parasitize both domestic and wild animals belong to the genera *Amblyomma*, *Rhipicephalus*, *Ixodes*, and *Hyalomma*, with these groups being the most widely distributed [71].

Research on tick distribution is crucial, as it facilitates the estimation of tick-borne disease prevalence and helps identify the ecological conditions in which ticks thrive [72]. Traditionally, the detection and identification of tick-borne pathogens have relied on culture-based, serological, and microscopic techniques [73]. However, due to the low sensitivity of culture methods, less than 2% of microorganisms can be successfully isolated under laboratory conditions [74].

Tick-borne pathogens are transmitted primarily through tick saliva, which serves as more than just a passive medium for pathogen delivery. Instead, the saliva of infected ticks comprises a complex mixture of hundreds of bioactive molecules that facilitate blood meal acquisition while ensuring physiological homeostasis [75].

Climate change has influenced tick distribution, with these ectoparasites now being observed in more northern regions, such as Canada and the Nordic countries, as well as at higher altitudes exceeding 1500 m. Conversely, their prevalence in southern regions may decline depending on species-specific adaptability. Increased awareness of vector-borne diseases among both healthcare professionals and the general public, alongside advancements in direct diagnostic methods—particularly molecular techniques such as PCR and culture—have contributed to improved disease management in both human and animal populations [76].

## 5. Laboratory Diagnosis

An overview of the primer sequences and diagnostic techniques utilized for the molecular identification of tick-borne diseases, with a focus on *Babesia* and *Anaplasma* species, is shown in Table 2. The table illustrates the variety of molecular targets and the methods used to guarantee precise pathogen identification and characterization in tick vectors, including nested PCR, real-time PCR, endpoint PCR, and conventional PCR (Polymerase Chain Reaction).

### 5.1. Laboratory Diagnosis in Babesiosis

#### 5.1.1. Microscopic Examination

Microscopic analysis of blood remains a conventional method for pathogen screening in infected animals. This technique involves staining blood smears using the Giemsa method or fluorescent dyes such as acridine orange. Examinations are conducted employing a 10× eyepiece and a 100× objective lens (with immersion oil). The detection sensitivity of this method allows for the identification of parasitemia levels as low as one parasite per 10^6^ red blood cells [100].

#### 5.1.2. PCR (Polymerase Chain Reaction)

The following figures are meant to illustrate the countries of origin of the reviewed studies and do not necessarily reflect the routine diagnostic practices implemented in those regions.

**Figure 2:**
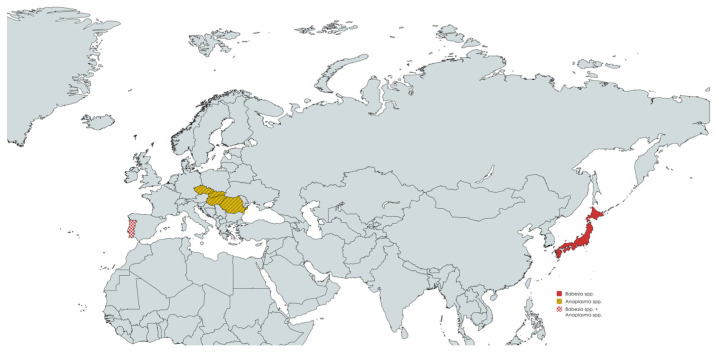
Geographic distribution of countries applying laboratory diagnostics for *Babesia* and *Anaplasma* spp. in wild boars [101].

Figure 2 illustrates the geographic distribution of countries where laboratory diagnostic methods targeting *Babesia* and *Anaplasma* spp. have been exclusively applied to samples collected from wild boars. The map offers a visual synthesis of current research coverage, highlighting regions actively involved in molecular surveillance of these pathogens.

In Japan, conventional and nested PCR targeting three genetic markers—the 18S rRNA gene, the internal transcribed spacer (ITS) region, and the cytochrome b (COB) gene were used to molecularly detect *Babesia* spp. in liver and whole blood samples taken from wild boars [102]. As explained by Hornok et al. (2014), primers BJ1 (5′-GTCTTGTAATTGGAATGATGG-3′) and BN2 (5′-TAGTTTATGGTTAGGACTACG-3′) were used in a screening conventional PCR that targeted a partial portion of the 18S rRNA gene [103]. For more detailed genotyping, a nested PCR approach targeting a longer fragment of the same gene was employed with two primer sets: BTH18S 1stF/1stR and BTH18S 2ndF/2ndR. Additionally, ITS region amplification was performed using primers ITS-F (5′-GAGAAGTCGTAACAAGGTTTCCG-3′) and ITS-2 (5′-ACAATTTGCGTTCAATCCCA-3′), and COB gene detection was carried out with primers COB-F (5′-CCATAGCAATTAATCCAGCTA-3′) and COB-R (5′-ACCTTGGTCATGGTATTCTG-3′) [104]. Agarose gel electrophoresis was used to examine each PCR product, and sequencing was used to validate the results. *Babesia* DNA from a variety of host tissues and geographic regions could be accurately detected and characterized thanks to the technology, which combined conventional and nested PCR [90].

The presence of *Babesia* spp. in wild boars from Portugal was evaluated using conventional PCR targeting the 18S rRNA gene, a conserved molecular marker for piroplasms. The reaction employed primers 5′-AATACCCAATCCTGACACAGGG-3′ and 5′-TTAAATACGAATGCCCCCAAC-3′, yielding a ~400 bp amplicon suitable for detection of both *Babesia* and *Theileria* spp. [104]. This method helped to better understand wildlife reservoirs in the epidemiology of tick-borne diseases in Portugal by enabling the genetic screening of protozoan infections of zoonotic and veterinary importance [92].

### 5.2. Laboratory Diagnosis in Anaplasmosis

#### PCR (Polymerase Chain Reaction) and RT-PCR (Reverse Transcription Polymerase Chain Reaction)

*Anaplasma phagocytophilum* was found in a research on wild boars in Slovakia using a real-time PCR (qPCR) experiment that targeted a 77 bp fragment of the msp2 gene using primers ApMSP2f (5′-ATGGAAGGTAGTGTGTTGGTTATGGTATT-3′), ApMSP2r (5′-TTGGTCTTGAAGCGCTCGTA-3′), and the TaqMan probe ApMSP2p (5′-TGGTGCCAGGGTTGAGCTTGAGATTG-3′), labeled with FAM [105]. Using a CFX96 Real-Time PCR System (Bio-Rad, USA), the reactions were carried out. Using conventional PCRs that targeted a 546 bp segment of the 16S rRNA gene, samples that showed positive results were further examined [106] and a 1297 bp fragment of the groEL gene [107] to assess genetic diversity. Positive control DNA originated from *A. phagocytophilum*-positive *Ixodes ricinus* ticks, while DNA-free water was used as a negative control. All positive amplicons were purified and subjected to bidirectional Sanger sequencing [96].

Conventional PCR was used in Portugal to identify *Anaplasma* spp. DNA in wild boars, employing several gene targets to improve species-level identification. The 16S rRNA gene was the focus of a first broad-range PCR using primers (5′-GGTACCYACAGAAGAAGTCC-3′) and (5′-TAGCACTCATCGTTTACAGC-3′), amplifying a 345 bp fragment [104]. Additionally, a groEL gene fragment (~600 bp) was amplified using primers 5′-ACTGATGGTATGCARTTTGAYCG-3′ and 5′-TCTTTRCGTTCYTTMACYTCAACTTC-3′ [108], a common marker for further molecular discrimination. For *A. phagocytophilum*, a more specific amplification targeted the msp4 gene, with primers ATGAATTACAGAGAATTGCTTGTAGG and TTAATTGAAAGCAAATCTTGCTCCTATG, yielding an 849 bp amplicon [46]. These findings validated the presence of zoonotic and general *Anaplasma* species in southern European wild boars [92].

Dreghiciu I.C. et al. (2023) evaluated the prevalence of *Anaplasma phagocytophilum* DNA in whole blood samples taken from wild boars from two areas in Romania. A total of 29 blood samples were analyzed using conventional PCR, targeting the epank1 gene of *A. phagocytophilum*. DNA was extracted using the ISOLATE II Genomic DNA Kit (Bioline), and PCR amplification was performed in 25 µL reactions using the MyTaq Red Mix Master Mix (Bioline). The primers used were LA6 (forward: 5′-GAG AGA TGC TTA TGG TAA GAC-3′) and LA1 (reverse: 5′-CGT TCA GCC ATC ATT GTG AC-3′), amplifying a 444 bp fragment. The PCR products were visualized by electrophoresis in a 1.5% agarose gel stained with Midori Green [78].

*Anaplasma phagocytophilum* was detected in tissue samples (spleen, liver, and kidney) from 870 wild boars in a molecular investigation carried out in Transylvania, Romania. iNtRON Biotechnology, Korea’s G-DEXTM IIC Genomic DNA Extraction kit was used to extract DNA. The 16S rRNA gene of *A. phagocytophilum* was the target of the nested PCR detection process. In the first round, a 932 bp fragment was amplified using primers ge3a (5′-CACATGCAAGTCGAACGGATTATTC-3′) and ge10r (5′-TTCCGTTAAGAAGGATCTAATCTCC-3′). The second (nested) round targeted a 546 bp internal fragment using primers ge9f (5′-AACGGATTATTCTTTATAGCTTGCT-3′) and ge2 (5′-GGCAGTATTAAAAGCAGCTCCAGG-3′), following the protocol described by Massung et al. (1998) [106]. PCR amplification was performed using a Bio-Rad iCycler, and amplicons were visualized by electrophoresis in 1.5% agarose gel stained with ethidium bromide. The total rate of *A. phagocytophilum* infection was 4.48%, with 2010–2012 showing a considerably higher rate (6.67%) than 2007–2008 (2.29%) [97].

The occurrence of *Anaplasma phagocytophilum* in wild ungulates, such as deer, water buffalo, and wild boars, was examined in a study carried out in Hungary. Real-time PCR targeting the msp2 gene was used to evaluate blood DNA samples. The detection assay utilized the primer pair ApMSP2f (5′-ATG GAA GGT AGT GTT GGT TAT GGT ATT-3′) and ApMSP2r (5′-TTG GTC TTG AAG CGC TCG TA-3′), along with a specific probe ApMSP2p (5′-TGG TGC CAG GGT TGA GCT TGA GAT TG-HEX-3′) [109].

The sensitive and precise detection of *A. phagocytophilum* was made possible by this real-time PCR technique, underscoring the possible reservoir role of big ungulates in the epidemiology of this zoonotic agent in Central Europe [94].

Blood samples from 550 wild boars collected as part of an African Swine Fever monitoring program were analyzed using molecular techniques for *Anaplasma phagocytophilum* and piroplasms in a study conducted in the Czech Republic. A nested PCR targeting a 407 bp fragment of the groEL gene was used to detect *A. phagocytophilum*. Positive samples were then subjected to phylogenetic analysis by amplifying a 1297 bp fragment of the groEL operon. DNA was extracted from blood, and PCR amplification was performed using 2x PCRBIO Taq Mix Red (PCR Biosystems, UK), with products visualized on a 1.5% agarose gel and sequenced for confirmation. Further analysis of the sequencing data was performed to search for phylogenetic linkages and haplotype differentiation [85].

In Wallonia, a region in Belgium, a molecular investigation was carried out during the 2011 hunting season (October to December) to assess the presence of *Anaplasma phagocytophilum* in wild boars. A total of 513 spleen samples were collected from hunted animals as part of a targeted wildlife health surveillance program. DNA extraction was performed from 50 mg of spleen tissue using the DNAzol reagent (Invitrogen), followed by quality and concentration assessment with a NanoDrop ND-1000 spectrophotometer. Detection of *A. phagocytophilum* relied on a nested PCR protocol targeting the 16S rRNA gene. The first round of PCR employed the broad-range primers EC9 (5′-TACCTTGTTACGACTT-3′) and EC12A (5′-TGATCCTGGCTCAGAACGAACG-3′), which amplify a 1462 bp fragment common to *Anaplasma* and *Ehrlichia* spp. The second round used the species-specific primers SSAP2F (5′-GCTGAATGTGGGGATAATTTAT-3′) and SSAP2R (5′-ATGGCTGCTTCCTTTCGGTTA-3′), targeting a 641 bp fragment unique to *A. phagocytophilum* [110]. Out of the 513 animals tested, 5 were found to be positive, resulting in a prevalence of 0.97%. Although the infection rate was low, this finding points to the silent circulation of *A. phagocytophilum* in wild boars from Wallonia and raises questions about the potential involvement of this species in local epidemiological cycles [99].

### 5.3. Laboratory Diagnosis in Tick Parasitism

*Anaplasma* species were found in ticks taken from wild boars in the Czech Republic by combining PCR and sequencing-based methods [111]. Broad-range identification of bacterial and protozoan vector-borne diseases was made possible by the PCR/ESI-MS approach; however, conventional PCR followed by Sanger sequencing was required to confirm *Anaplasma* at the species level. Specifically, two primer sets were used to amplify *Anaplasma*-specific targets: Ap-msp4-F (M13F-ICAGTMTGYGCYTGCTCCCT)/Ap-msp4-R (M13R-CCTTAIYTGAAMISIAATCTTGCTCC) targeting the msp4 gene, and Ap-groEL-F (M13F-GAIAIIACTGAYGGTATGCAGTTTG)/Ap-groEL-R (M13R-CYAIMCIYTCYYTMAGYTTTTCCTT) targeting the groEL gene. These reactions were performed using high-fidelity Taq polymerase and analyzed by agarose gel electrophoresis followed by sequencing. Reliable amplification for subsequent sequence-based characterisation was made possible using this conventional PCR technology, which is not real-time. The study emphasized the spread of several *Anaplasma* strains in Central European ticks that parasitize wild boars [85].

A study conducted in the southern region of the Czech Republic investigated the presence of vector-borne pathogens in *Ixodes ricinus* ticks using a broad-range polymerase chain reaction coupled with electrospray ionization mass spectrometry (PCR/ESI-MS) platform. This method integrated both multiplex and singleplex PCR reactions targeting conserved bacterial and protozoan genes. For the detection of *Babesia* spp., two primer pairs were employed: INV10812F/INV10813R targeting the β-tubulin gene (TGAGAGAAATCGTACACATTCAAGCGGG/TCCATGTTCGTCGGAGATGACTTCCCA) and INV10034F/INV10035R targeting the 18S rRNA gene (TGCGCAAATTACCCAATCCTGACAC/TCCAGACTTGCCCTCCAATTGGTA) as reported by Crowder et al. (2012) [111]. Additionally, confirmatory sequencing was performed using M13-tagged primers targeting 18S rRNA, ITS1, ITS2, and hsp70 gene regions [112]. For the identification of *Anaplasma* spp., sequencing primers were designed to amplify fragments of the msp4 gene (M13F-ICAGTMTGYGCYTGCTCCCT/M13R-CCTTAIYTGAAMISIAATCTTGCTCC) and the groEL gene (M13F-GAIAIIACTGAYGGTATGCAGTTTG/M13R-CYAIMCIYTCYYTMAGYTTTTCCTT). DNA was extracted from field-collected *I. ricinus* ticks, and internal positive controls using synthetic DNA were included to ensure amplification fidelity. This advanced technique can identify a wide range of tick-borne infections from vector samples using a large-scale, multiplex-compatible diagnostic platform [95].

Ticks taken from wild boars in Hungary were shown to be *A. phagocytophilum* carriers using a TaqMan real-time PCR technique that targets the msp2 gene, which amplifies a 77 bp area. The primers and probe used were based on the protocol of Courtney et al. (2004) [105], with the probe sequence modified as 5′-6-FAM-TGGTGCCAGGGTTGAGCTTGAGATTG-TAMRA-3′. Samples with a Ct value below 39 were considered positive. To assess strain diversity, all positive samples were further subjected to conventional PCR targeting an approximately 600 bp fragment of the groEL gene, using primers EphplGroEL(569)F (5′-ATGGTATGCAGTTTGATCGC-3′) and EphGroEL(1142)R (5′-TTGAGTACAGCAACACCACCGGAA-3′), following the method described by Alberti et al. (2005) [113]. These assays were performed on DNA extracted from ticks removed from wild boars, not from host tissue [80].

In the same study from Hungary, the presence of *Babesia* spp. DNA was screened in the population of ticks removed from peri-urban wild boars using a conventional PCR assay targeting a ~500 bp fragment of the 18S rRNA gene, following a protocol modified from Casati et al. (2006) [114]. The primers used were BJ1 (5′-GTCTTGTAATTGGAATGATGG-3′) and BN2 (5′-TAGTTTATGGTTAGGACTACG-3′). The PCR was performed in 25 μL reaction volumes with 1 μM of each primer and 1 U of HotStarTaq Plus DNA polymerase. Amplified products were visualized via agarose gel electrophoresis. These analyses were also conducted on DNA extracted from ticks, not directly from wild boar tissues. Among the positive findings, *Babesia canis* was detected in *Dermacentor reticulatus*, and zoonotic *Babesia* cf. *crassa* was identified in *Haemaphysalis concinna*, marking the first report of this species in Hungary [80].

A study conducted in Turkey using conventional PCR, which targets the 18S rRNA gene, reported *Babesia* spp. in ticks collected from wild animals. An initial PCR screening was carried out using BJ1 (5′-GTC TTG TAA TTG GAA TGA TGG-3′) and BN2 (5′-TAG TTT ATG GTT AGG ACT ACG-3′) primers, amplifying a fragment of 411–452 bp, under cycling conditions of 40 cycles at 94 °C for 30 s, 55 °C for 30 s, and 72 °C for 1 min. Samples that tested positive were further subjected to amplification of the near full-length 18S rRNA (~1700 bp) using primers Nbab_1F and TB Rev, applying 40 cycles with 2 min elongation at 72 °C to ensure sufficient coverage. This dual-step molecular approach enabled enhanced resolution in detecting *Babesia* genotypes in field-collected ticks from various wild hosts [91].

Detection of *A. phagocytophilum* DNA was achieved using conventional PCR targeting the msp4 gene, with MAP4AP5 (forward) and MSP4AP3 (reverse) primers, which amplified an 849 bp fragment. PCR conditions included 40 cycles of amplification at 94 °C for 30 s, 54 °C for 30 s, and 72 °C for 1 min, followed by a final elongation at 72 °C for 10 min. DNA was extracted from ticks removed from wild animals using standard protocols and tests performed confirmed the presence of *A. phagocytophilum* in tick populations parasitizing wildlife in natural Turkish ecosystems [91].

A study carried out genetic monitoring of tick-borne pathogens, such as *A. phagocytophilum* and *Babesia* spp., in questing and feeding hard ticks that were collected from wild hosts and vegetation in Northwestern Spain. DNA was extracted from individual adult ticks and pooled nymphs using the DNeasy Blood and Tissue Kit (Qiagen), and pathogen detection was carried out using both real-time PCR and conventional PCR methods. For *A. phagocytophilum*, real-time PCR targeted the msp2 gene, using the primers ApMSP2-FN1 (5′-AAGGCAGTGTTGGKTAYGGTATT-3′), ApMSP2-R (5′-TTGGTCTTGAAGCGCTCGTA-3′), and a Cy5-labeled probe (5′-Cy5-TGGTGCCAGGGTTGAGCTTGAGATTG-BHQ3-3′) [105]. Additional confirmation and variant typing were performed via nested PCR targeting the 16S rRNA gene, using the primer sets ge3a/ge10r and ge9f/ge2 [81,106].

For the detection of piroplasms, including *Babesia* spp., conventional PCR targeted the 18S rRNA gene using the primer pair BJ1 (5′-GTCTTGTAATTGGAATGATGG-3′) and BN2 (5′-TAGTTTATGGTTAGGACTACG-3′), amplifying a ~500 bp region. These primers are widely used for broad detection of *Babesia* and *Theileria* species [115]. All real-time reactions were run on a StepOne Plus platform (Applied Biosystems), while endpoint PCR was performed on a 2720 Thermal Cycler.

The significance of vector monitoring in endemic areas of Spain is highlighted by this study, which shows that both questing and feeding ticks from wildlife-associated habitats carry zoonotic tick-borne agents [81].

The presence of tick-borne infections in ticks taken from wild boars was examined in a study carried out in Romania, in Southern Europe. To find *A. phagocytophilum* and *Babesia/Theileria* spp., DNA was taken from the ectoparasites and analyzed using standard PCR tests [79].

For *A. phagocytophilum*, a nested PCR targeting the 16S rRNA gene was employed using primers Ge3a (5′-CACATGCAAGTCGAACGGATTATTC-3′) and Ge10 (5′-TTCCGTTAAGAAGGATCTAATCTCC-3′) in the first round, followed by Ge2 (5′-AACGGATTATTCTTTATAGCTTGCT-3′) and Ge9 (5′-GGCAGTATTAAAAGCAGCTCCAGG-3′) in the second round [106]. To further characterize the strains, groEL gene amplification was performed using EphplgroEL(569)F, EphplgroEL(1193)R, or EphgroEL(1142)R as primers [113].

For *Babesia/Theileria* spp., detection was based on amplification of a fragment of the 18S rRNA gene using the primer pair BJ1 (5′-GTCTTGTAATTGGAATGATGG-3′) and BN2 (5′-TAGTTTATGGTTAGGACTACG-3′) [116].

These results emphasize the importance of ticks in the ecology of tick-borne diseases in peri-urban environments by confirming that ticks that parasitize wild boars may act as zoonotic pathogen vectors [79].

An investigation of the occurrence of *A. phagocytophilum* was conducted in Northeastern Poland using ticks collected from wild ungulates, such as red deer and wild boars. DNA extracted from individual ticks was screened through conventional PCR, targeting a 247 bp fragment of the 16S rRNA gene. Detection was performed using the EHR521 (5′-TGTAGGCGGTTCGGTAAGTTAAG-3′) and EHR747 (5′-GCATCCTCATCCTTTACAGCGTG-3′) primer pair following the protocol described by Michalski et al. [117,118].

The potential danger of pathogen circulation in the sylvatic environment of northern Poland was highlighted by the sensitive detection of *A. phagocytophilum* DNA in tick vectors parasitizing free-ranging ungulates made possible by this PCR-based method [87].

In a study carried out in Sweden, *A. phagocytophilum* was screened in ticks collected from wild ungulates (including wild boars), using molecular methods focused exclusively on tick samples. DNA was extracted from non-engorged (questing) ticks using an ammonium hydroxide protocol, while engorged ticks were processed with the Qiagen DNeasy Blood & Tissue Kit (Qiagen, Hilden, Germany), following the manufacturer’s instructions. Lysates were stored at 4 °C until further analysis. Detection of *A. phagocytophilum* was performed using a duplex real-time PCR (qPCR) assay that also included Borrelia burgdorferi sensu lato as a co-target, though only *A. phagocytophilum* results were reported. The qPCR targeted the msp2 gene, using ApMSP2f (5′-ATG GAA GGT AGT GTT GGT TAT GGT ATT-3′) and ApMSP2r (5′-TTG GTC TTG AAG CGC TCG TA-3′) as primers, along with the ApMSP2p-[HEX]TGG TGC CAG GGT TGA GCT T probe. The protocol complied with the method described by Allender et al. and was implemented at the Centre for Ecological and Evolutionary Synthesis (CEES), University of Oslo [119]. By focusing on both engorged and questing ticks collected from wildlife, the study provided valuable insight into the circulation of *A. phagocytophilum* in tick populations across Swedish habitats.

### 5.4. Laboratory Diagnosis in Wild Boar and Associated Ticks for Both Pathogens (Babesia spp. and Anaplasma spp.)

**Figure 3:**
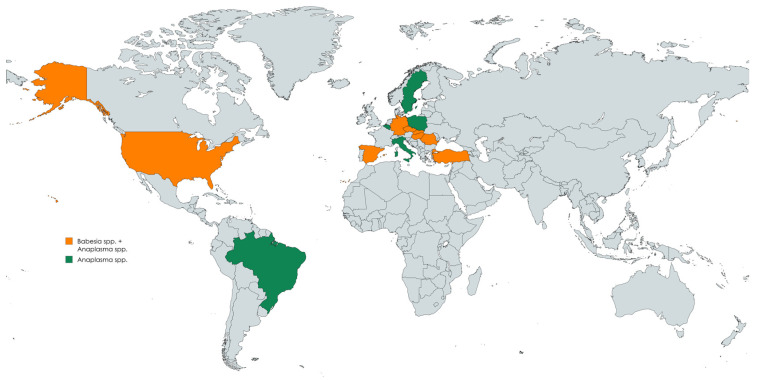
Geographic distribution of diagnostic studies in ticks and wild boar tissues [101].

Countries where molecular diagnostic techniques have been used to identify *Babesia* and/or *Anaplasma* spp. in ticks collected from wild boars and/or directly from wild boar tissues are represented geographically in Figure 3. The map identifies nations that have carried out laboratory-based research; orange countries have been investigated for both infections, whereas green countries have only researched *Anaplasma* spp.

These results highlight areas that are actively advancing our knowledge of the ecology of tick-borne diseases in wildlife and demonstrate the widespread interest in pathogen surveillance within wild boar-vector systems.

The presence of *Babesia* spp. DNA in wild ungulates and their associated ticks in Slovakia was evaluated using both conventional PCR and quantitative duplex PCR assays targeting multiple gene regions [93]. A conventional PCR was applied to amplify a ~450 bp fragment of the 18S rRNA gene, described by Casati et al. [114] and Hamšíková et al. [120]. Additionally, a duplex qPCR approach was used to simultaneously amplify a 62 bp fragment of the 18S rRNA gene and a 104 bp fragment of the internal transcribed spacer (ITS) region [121]. These techniques demonstrated the epidemiological complexity of piroplasm infections in Slovak wildlife by enabling both species-level and broad-range screening for *Babesia* in a variety of tissues and tick stages [93].

The molecular detection of *Anaplasma* spp. in wild boars and their associated ticks has been carried out using conventional PCR protocols targeting the 16S rRNA gene. In a study conducted in Brazil by Santana et al. (2022) [84], a single-round PCR was performed using the primer pair EHR16SD (5′-GGTACCYACAGAAGAAGTCC-3′) and EHR16SR (5′-TAGCACTCATCGTTTACAGC-3′), which amplifies a ~345 bp fragment of the 16S rRNA gene conserved among *Anaplasmataceae*. PCR products were separated by agarose gel electrophoresis and stained with ethidium bromide for visualization under UV light. Positive and negative controls were included in each run to validate amplification specificity and rule out contamination. This conventional PCR approach has been widely used in epidemiological surveillance of *Anaplasma* spp. in wildlife reservoirs and arthropod vectors [84].

Real-time PCR and nested PCR methods were used in Slovakia to detect *A. phagocytophilum* in free-ranging animals, such as wild boars, and in *I. ricinus* ticks [93]. The initial screening was based on real-time PCR targeting a 77 bp fragment of the msp2 gene, according to the protocol of Courtney et al. [105]. For deeper genotypic characterization, selected positive samples from host tissues and engorged larval ticks were further subjected to nested PCR amplification of a 546 bp fragment of the 16S rRNA gene [106,122], as well as qPCR targeting a 530 bp fragment of the groEL gene [113]. This multilocus approach, combining high-sensitivity qPCR with classical nested PCR, enhanced both detection and phylogenetic resolution of circulating *A. phagocytophilum* strains in sylvatic ecosystems of Slovakia [93].

In Northeastern Italy, *Anaplasma phagocytophilum* was investigated in wild boar blood samples and associated ticks using both real-time PCR and conventional PCR techniques. DNA was extracted from 200 µL of whole blood and from tick homogenates using the DNeasy Blood & Tissue Kit (QIAGEN). An internal DNA control was spiked into each sample to detect PCR inhibition. Screening for *A. phagocytophilum* was performed by TaqMan real-time PCR targeting a 77 bp region of the msp2 gene, using the primers ApMSP2f (5′-ATGGAAGGTAGTGTTGGTTATGGTATT-3′), ApMSP2r (5′-TTGGTCTTGAAGCGCTCGTA-3′), and the probe ApMSP2p (5′-HEX-TGGTGCCAGGGTTGAGCTTGAGATTG-BHQ1-3′), as described by Courtney et al. [105]. Amplifications were conducted on a LightCycler96 platform, and samples with Cq values ≤ 40 were considered positive. For strain characterization, a conventional PCR targeting a ~600 bp region of the groEL gene was performed using primers groEL643f (5′-ACTGATGGTATGCARTTTGAYCG-3′) and groEL1236r (5′-TCTTTRCGTTCYTTMACYTCAACTTC-3′), following the method of Alberti et al. [113]. Amplification products were visualized on 2% agarose gel and sequenced bidirectionally. The results revealed that zoonotic ecotypes of *A. phagocytophilum* circulate among wild ungulates and their ectoparasites in this region, confirming the potential epidemiological role of wild boars in the transmission cycle of this pathogen [89].

A molecular study conducted in Southern Germany evaluated the prevalence of *Babesia* spp. and *A. phagocytophilum* in engorged *I. ricinus* ticks and wild boars. Between 2010 and 2013, spleen (*n* = 24), blood (*n* = 21), and skin (*n* = 12) samples were collected from 24 professionally hunted wild boars in the Angelberger Forst region, along with 16 engorged ticks retrieved from two animals. DNA was extracted from all tissues and tick samples and tested for pathogens using conventional PCR.

For *A. phagocytophilum*, a conventional PCR targeting the 16S rRNA gene was performed, followed by sequencing of the amplicons. While exact primer sequences were not listed in this article, the authors refer to previous methods [123], likely employing the primer sets ge3a/ge10r and ge9f/ge2 [106], commonly used for this target.

Detection of *Babesia* spp. was also conducted via conventional PCR, using DNA extracted from blood, spleen, and ticks, amplifying a region of the 18S rRNA gene. Based on related methodologies cited by the authors, the primers BJ1 (5′-GTCTTGTAATTGGAATGATGG-3′) and BN2 (5′-TAGTTTATGGTTAGGACTACG-3′) were most likely used, as described in Casati et al. (2006) [114].

This study highlighted the co-circulation of zoonotic tick-borne pathogens in both host and vector, reinforcing the potential role of wild boars as reservoir hosts in Central Europe [98].

In a research carried out in Guam, USA, both wild pigs and the ticks that they were associated with were tested for tick-borne diseases using standard PCR techniques. Whole blood samples from wild pigs and tick homogenates were used to obtain DNA. In blood samples from wild pigs, *Anaplasma platys* was detected using primers PLATYS-F (5′-TTGATTTTTGTCGTAGCTTGCT-3′) and PLATYS-R (5′-TTGATTTCTCTCATTCCCCGT-3′), which amplify a 349 bp fragment of the 16S rRNA gene. Additionally, *B. canis vogeli* was identified in wild pig blood using primers BTF1 (5′-TTGGCAAGGAATTAAAACTCCTTTG-3′) and BTR2 (5′-CTAAGAATTTCACCTCTGACAGT-3′), targeting the 18S rRNA gene.

In contrast, *A. phagocytophilum* and *Ehrlichia canis* were detected in ticks removed from wild pigs. For *A. phagocytophilum*, primers ApMSP2f (5′-ATG GAA GGT AGT GTT GGT TAT GGT ATT-3′) and ApMSP2r (5′-TTG GTC TTG AAG CGC TCG TA-3′) targeting the msp2 gene were used. All PCR assays were conventional (endpoint) and amplicons were visualized by gel electrophoresis.

These findings highlight the co-circulation of multiple tick-borne agents in both wild pigs and their ectoparasites in a tropical island ecosystem [82].

In a four-year wildlife surveillance study conducted in the Liguria region of Northwest Italy, *Anaplasma* spp. were detected using endpoint PCR targeting the 16S rRNA gene. DNA was extracted from both blood samples of wild boars and from associated tick homogenates. For initial screening, the primers 16SANA-F (5′-CAGAGTTTGATCCTGGCTCAGAACG-3′) and 16SANA-R (5′-GAGTTTGCCGGGACTTCTTCTGTA-3′) were used [124]. Confirmatory PCR amplifications of *Anaplasma* spp. also targeted the 16S rRNA gene, using the primer sets EE1 (5′-TCCTGGCTCAGAACGAACGCTGGCGGC-3′), EE2 (5′-AGTCACTGACCCAACCTTAAATGGCTG-3′), EE3 (5′-GTCGAACGGATTATTCTTTATAGCTTGC-3′), and EE4 (5′-CCCTTCCGTTAAGAAGGATCTAATCTCC-3′), as described by Richter et al. [125].

Agarose gel electrophoresis was used to visualize the amplicons of all traditional (end-point PCR) amplifications, which were performed using PlatinumTM Taq DNA Polymerase or GoTaq G2 DNA Polymerase. Sequences of the positive samples were matched to GenBank sequences to confirm the species. The significance of combined host-vector surveillance is underscored by these findings, which support the circulation of *Anaplasma* species in wild boars and the ticks that are associated with them in a Mediterranean environment [77].

In Poland, the detection of *A. phagocytophilum* DNA in spleen and blood samples from wild boars, as well as in attached *I. ricinus* ticks, was conducted using conventional and nested PCR protocols [88]. For initial screening, a conventional PCR targeting the msp2 gene was performed, using primer sequences listed in Table 2 of the study. To confirm and sequence the obtained amplicons, a nested PCR approach was applied, targeting the groEL operon using two primer sets: HS1/HS6 and HS43/HS45. The PCR reactions followed thermal cycling conditions previously described by Massung and Slater (2003) [126]. The amplified products were analyzed via 2% agarose gel electrophoresis and visualized under UV light following ethidium bromide staining. Positive samples were selected for sequencing to assess genetic variation and homology with known *A. phagocytophilum* strains. This strategy, combining classical PCR and nested PCR, proved effective in detecting *A. phagocytophilum* in both host and vector samples under field conditions [88].

In a study conducted by Castillo-Contreras et al. (2022) [83] in the metropolitan area of Barcelona, Spain, a total of 167 spleen samples from wild boars (*Sus scrofa*) and 180 tick pools collected from these animals were analyzed for the presence of vector-borne pathogens. Detection of *Anaplasma* spp. and *Ehrlichia* spp. was performed using conventional PCR followed by reverse line blot (RLB) hybridization. Primers targeted the 16S rRNA gene: 16S8FE (5′-GGAATTCAGAGTTGGATC(A/C)TGG(C/T)TCAG) and BGA1B-new (5′-Biotin-CGGGATCCCGAGTTTGCCGGGACTT(C/T)TTCT), amplifying a fragment of 460–520 bp. For the detection of *Babesia* spp., conventional PCR followed by RLB was also employed, targeting the 18S rRNA gene with primers RLB-F2 (5′-GACACAGGGAGGTAGTGACAAG) and RLB-R2 (5′-Biotin-CTAAGAATTTCACCTCTGACAGT), yielding an expected product size of 460–540 bp. All protocols followed the methodology described by Lorusso et al. (2016) and included appropriate positive and negative controls to ensure assay reliability [83,127].

Multiple pathogens may be simultaneously detected and genotyped from tissue and ectoparasite samples thanks to these tests. The significance of this host species in the ecology of tick-borne diseases in peri-urban European settings was highlighted by the detection of mixed infections in the wild boar-tick system using RLB, a traditional PCR-based technique [83].

## 6. Discussion

According to the reviewed studies, molecular diagnostics remain the predominant method reported for identifying tick-borne pathogens in wild boar and associated ectoparasites, with classical PCR and real-time PCR being the most frequently employed techniques. These approaches offer high specificity and sensitivity and have enabled the detection of a wide array of pathogens such as *Anaplasma phagocytophilum*, *Babesia* spp., and *Theileria* spp. across multiple sample types, including blood, spleen, liver, and engorged ticks. Despite the extensive use of PCR-based protocols, our literature review reveals a distinct gap regarding serological methods.

Sampling wild boar populations inevitably involves logistical and ethical considerations, including maintaining carcass freshness, complying with seasonal hunting regulations, and minimizing sampling bias, which may vary depending on each country’s wildlife management and animal welfare standards. These factors can influence both the quality of collected samples and the representativeness of diagnostic results.

Notably, none of the studies evaluated from 2005 to the present have used ELISA, a commonly used technique in domestic animal and human diagnostics, to detect *Babesia* or *Anaplasma* infections in wild boars or their ectoparasites. In many instances, the study objectives were ecological or epidemiological trends rather than illness diagnosis in general, or the diagnostic techniques were not clearly defined. This shortcoming emphasizes the necessity of focused serological investigations to supplement molecular methods and offer a more comprehensive comprehension of pathogen exposure dynamics and host immune responses in wild boar populations.

A possible sampling bias could result from the overrepresentation of studies performed in Central and Eastern Europe, while fewer studies were available from other regions such as Western Europe, Asia outside Japan, or the Americas. In Western Europe, wild boar populations are also absent in some regions, such as Ireland and the United Kingdom, where only recent controlled reintroductions exist. This geographic imbalance may reflect differences in research focus, available funding, or publication language. Other factors should also be considered, such as the presence and distribution of wild boar populations, which are absent from large areas of the American continent or have a recent colonization history in some regions of Latin America. Additionally, differences in wild boar density, hunting practices, and tick surveillance programs among countries might have influenced the number and type of studies included.

There is a difference in methodological approaches and pathogen detection rates between nations when examining diagnostic techniques employed over the previous 20 years to identify *Babesia* spp. and *Anaplasma* spp. in wild boars and their associated ticks. These variations depend on several factors, including infrastructure availability, research focus, and the order of importance of zoonotic surveillance programs, in addition to geographic dispersion.

Molecular diagnostic methods like nested PCR that target the groEL and 16S rRNA genes have been widely used in Germany. By using these molecular techniques consistently across blood, spleen, and skin tissue types, researchers have been able to describe the co-occurrence of several infections, such as *Anaplasma phagocytophilum* and *Babesia* spp. Southern studies highlighted a possible zoonotic danger, particularly in peri-urban areas where domestic animals and wild boars come in contact [98].

Studies from Hungary showed a stronger focus on ecotyping and genetic characterization of *A. phagocytophilum* strains. Using both real-time PCR and conventional PCR protocols (targeting *msp2* and *groEL*), several studies aimed to explore the zoonotic potential and phylogenetic distribution of circulating strains in wild ungulates. The use of sequence-confirmed positive controls has bolstered the credibility of results [80].

Italy stands out through its implementation of both conventional and real-time PCR, with some of the most technically diverse studies. Italian researchers frequently targeted multiple genes simultaneously (e.g., *msp2*, *groEL*, and 16S rRNA) and included controls for potential PCR inhibition. Moreover, co-infections with other TBPs such as *Rickettsia* spp. and *Borrelia burgdorferi s.l.* were often considered, particularly in studies involving ticks from wild boars in northern regions [77].

Studies conducted in Romania have shown a steady rise in *A. phagocytophilum* detection rates, especially during the second half of the studied period (2010–2012). The primary diagnostic technique, nested PCR, mainly targeted the 16S rRNA gene. Regional prevalence comparisons were made possible by a more centralized sampling technique spanning many counties, demonstrating an epidemiological dynamic in central Transylvania and northwest areas [78,79].

Spain and Portugal used more diverse strategies, frequently integrating both PCR and serological techniques. In Spain, reverse line blot (RLB) hybridization was used alongside real-time PCR for a broader screening approach. This combination increased the detection sensitivity for mixed infections in both ticks and wild boar tissues. Portugal’s studies also focused on the specificity of primers used, especially when differentiating *Anaplasma* species, such as *A. marginale*, *A. ovis*, and *A. centrale*, using gene targets like *msp4* and 16S rRNA [83,92].

Real-time PCR dominated the molecular diagnostics scene in Slovakia and the Czech Republic, especially for the identification of *A. phagocytophilum* through the msp2 gene. Deeper molecular characterisation was made possible by the frequent use of groEL or 16S rDNA fragment sequencing in addition to PCR in these investigations. Following ASF monitoring efforts, the Czech data particularly benefited from a high sample volume, which produced an uncommon large-scale dataset for TBP screening in wild boars [85,93,95].

Overall, this cross-country study shows a common tendency in addition to methodological heterogeneity: the preponderance of PCR-based molecular techniques for TBP detection. One significant knowledge gap is the lack of ELISA or serologically based diagnostics in these nations, particularly for *Babesia* and *Anaplasma* in wild boars and associated ticks. The limitations of ELISA’s sensitivity and specificity for animals, together with practical and moral restrictions in sample collection and validation, are probably the cause of this discrepancy.

Due to the relatively recent development and expansion of wild boar populations as invasive species, research on tick-borne infections (TBPs) in wild boars in the United States has been limited compared to European nations. However, using PCR techniques that target genes like msp2 and 18S rRNA, molecular investigations carried out in places like Guam and areas of the southern U.S. have found *Anaplasma phagocytophilum* and *Babesia* spp. in blood samples. The use of real-time PCR was common, employing TaqMan probes for enhanced sensitivity, especially in studies involving multi-species surveillance, including domestic animals, wild pigs, and environmental tick sampling. Despite the advancements in molecular tools, serological methods such as ELISA remain largely unreported in wild pig populations, likely due to the focus on direct pathogen detection and the lack of standardized serological kits validated for wildlife [82].

In Japan, the focus has been more ecologically and genetically oriented, with studies emphasizing the phylogenetic diversity of *Anaplasma* and *Babesia* species in both wild boars and their tick vectors. Molecular diagnostics, including nested PCR and sequencing of *groEL*, *msp4*, and 16S rRNA genes, have been the cornerstone of detection strategies. Real-time PCR assays targeting the *msp2* gene were also employed for *A. phagocytophilum* detection. Notably, similar to trends observed in Europe and the U.S., no studies were identified in Japan that used ELISA for the diagnosis of babesiosis or anaplasmosis in wild boars or their ectoparasites. This may reflect both the challenges in developing reliable serological assays for wildlife and a research focus more oriented toward molecular epidemiology and tick–host–pathogen dynamics [90].

The low prevalence (0.97%) of *Anaplasma phagocytophilum* detected in wild boars from Belgium may reflect either a limited exposure to infected ticks in that region or a transient bacteremia that reduces detection rates in spleen tissue. The use of a nested PCR targeting the 16S rRNA gene increased the sensitivity of detection, yet the relatively small number of positive cases suggests that wild boars may play a minor role as reservoir hosts in this ecosystem. Additionally, given the temporal and geographic constraints of the sampling (limited to the 2011 hunting season), the results might underestimate broader seasonal or interannual variation in pathogen circulation [99].

In contrast, the Swedish study focused on *Ixodes ricinus* ticks collected from wild ungulates and questing in the environment, employing a duplex real-time PCR targeting the *msp2* gene of *A. phagocytophilum*. This approach allowed for high-throughput screening and detection of the pathogen directly in the vector population. Unlike host-based surveys, tick-focused sampling provides a broader ecological snapshot of pathogen prevalence and circulation. However, without direct linkage to infected hosts, such findings cannot fully clarify the reservoir competence of specific ungulate species, though they offer valuable insights into exposure risk and vector–pathogen dynamics in Swedish habitats [86].

Monitoring animal diseases using molecular and serological diagnostics can significantly support a One Health concept. Veterinary and public health treatments can be supported and zoonotic pathogen surveillance improved by the systematic collection and analysis of diagnostic data from wild boars and the ticks that carry them. Across the human–animal–environment interface, standardized procedures and coordinated data exchange among public health officials, wildlife managers, and veterinarians may aid in mapping the distribution of pathogens, identifying new threats, and bolstering preventative measures.

Overall, the reviewed studies confirm that both conventional and real-time PCR have been applied extensively for detecting *Babesia* and *Anaplasma* infections in wild boars and their ticks. Conventional PCR has the advantage of lower technical requirements and costs, making it accessible for smaller laboratories or regions with limited resources. In contrast, real-time PCR demonstrates superior sensitivity, faster processing, and quantitative capabilities, which are crucial when screening large numbers of samples or detecting low pathogen loads. Nested PCR was also adopted to enhance sensitivity, especially in samples with poor DNA quality or low parasitemia, but comes with a higher contamination risk due to multiple amplification rounds. While each method offers reliable pathogen detection, their comparative performance depends on the sample matrix (blood, spleen, ticks), local laboratory infrastructure, and the intended goal (prevalence monitoring versus genetic characterization).

The differences in preferred molecular methods across countries reflect not only research priorities but also practical and economic considerations. For example, real-time PCR was more frequently applied in Western European countries, where investment in equipment and trained personnel is more feasible. In Eastern Europe, conventional or nested PCR remained more common, partly due to its affordability and ease of implementation in routine wildlife surveillance. The selection of specific primers also varied according to the regional focus on zoonotic threats or co-infections of veterinary concern. These patterns underline the importance of considering local infrastructure, costs of reagents, and technical know-how when interpreting published results or designing future monitoring programs.

Although serological techniques such as ELISA are routinely used in domestic animals and humans, none of the reviewed studies employed ELISA for wild boar or tick samples. This gap can be explained by multiple factors: lack of commercial kits validated for wildlife, uncertainty regarding antibody kinetics in free-ranging populations, and the ethical and logistical hurdles of collecting sufficient serum samples for validation studies. In addition, regulatory requirements for wildlife diagnostics are less standardized than those for domestic species, creating further obstacles. Developing and validating serological assays specifically for wildlife hosts could fill this gap, complementing molecular methods by revealing past exposure and improving our understanding of infection dynamics in these populations.

Few commercial ELISA kits have been validated for use in wildlife. Interpreting antibody responses in free-ranging populations can be challenging, or molecular diagnostic techniques like PCR, which provide higher specificity and sensitivity in identifying active infections, are generally preferred. These factors may contribute to the absence of serological data in these host species. This deficiency highlights the need for further research into the applicability and reliability of serological tools like ELISA in the epidemiological surveillance of *Babesia* and *Anaplasma* spp. in wild boar populations and their associated ectoparasites.

Point-of-care molecular or immunodiagnostic assays could represent a promising adjunct to traditional laboratory-based techniques, offering faster results and enhancing wildlife surveillance capacities, especially in remote or resource-limited settings.

The two-step method of initial molecular screening using real-time PCR assays targeting the 16S rRNA or msp2 gene for *Anaplasma* spp. and the 18S rRNA gene for *Babesia* spp., followed by confirmatory sequencing of positive samples to ensure species-level resolution, could be the basis of a practical diagnostic framework for field surveillance in wild boars, according to the reviewed studies. To more accurately describe exposure status, approved serological tests may be used in conjunction with these molecular techniques when they are available. By using such standardized procedures, wildlife disease monitoring systems may be strengthened, and data comparability across areas may be enhanced.

Currently, no standardized or mandatory surveillance programs for *Babesia* and *Anaplasma* spp. in wild boar populations exist at the European or global level. Most monitoring efforts have been research-based and localized, often depending on funding availability or academic initiatives. This highlights a significant policy gap and suggests the need for coordinated surveillance frameworks to better track these zoonotic pathogens and inform both wildlife and public health strategies.

Over the past two decades, diagnostic approaches for *Babesia* spp. and *Anaplasma* spp. in wild boars and their ticks have advanced considerably. Initially, studies relied mostly on conventional PCR with broad-range primers, but more recent investigations have increasingly adopted real-time PCR assays with refined primer designs and probe-based detection, achieving higher sensitivity and specificity. This shift reflects both technological progress and a greater demand for reliable, rapid diagnostics to support wildlife disease surveillance.

Given the diversity of methods found in the reviewed literature, there is a clear need to establish more standardized diagnostic protocols for monitoring tick-borne pathogens in wild boars and their ticks. PCR with validated primers should be prioritized for detecting active infections, while serological testing could complement these approaches if reliable assays for wildlife become available. Additionally, harmonizing sample collection procedures, including tissue selection, storage conditions, and timing, would improve the consistency and comparability of data across different studies and regions.

## 7. Conclusions

According to the reviewed literature, the most accurate techniques for identifying *Anaplasma* and *Babesia* spp. in wild boars and the ticks that accompany them are the molecular methods, specifically conventional PCR, nested PCR, and real-time PCR. Genetic markers, including msp2, groEL, 16S rRNA, and 18S rRNA, were often utilized for pathogen identification in studies conducted in several European nations, including Slovakia, Portugal, Italy, Spain, Germany, and the Czech Republic. Real-time PCR was frequently chosen because of its favorable diagnostic characteristics, including high sensitivity, specificity, and overall accuracy. Notably, while diagnosing these infections in wild boars, serological techniques like ELISA were virtually nonexistent.

Given these gaps, future research should focus on developing and validating wildlife-specific serological assays, standardizing molecular diagnostic protocols for better cross-study comparability, and conducting studies that evaluate the cost-effectiveness and feasibility of these diagnostic tools in field conditions. This would strengthen the surveillance and management of tick-borne diseases in wild boar populations and their associated ectoparasites.

Relevant molecular research was also carried out outside of Europe in nations like the USA and Japan, with emphasis on strain characterization and DNA-based detection. There was no indication that wild boar or tick samples had been subjected to ELISA or other serological techniques, even in these areas.

In the context of the reviewed studies, serological testing seems to be less commonly applied, possibly due to the lack of validated assays for wild species or because exposure-based surveillance receives less attention in these research settings.

## Figures and Tables

**Table 1:** Criteria for exclusion of studies.

Exclusion Criteria	Description
Wrong host species	Studies conducted on animals other than wild boars
Wrong pathogen	Studies targeting pathogens other than *Babesia* spp. or *Anaplasma* spp.
Wrong diagnostic method	Studies using only serology, microscopy, or culture without molecular confirmation
No molecular diagnostic data	Articles describing only epidemiology or tick ecology without molecular methods
Non-original study	Reviews, letters, or opinion papers without primary data
Language other than English	Studies published in other languages
No full-text available	Abstracts, theses, or papers with no full text available
Duplicate publication	Duplicate records retrieved from multiple databases
Insufficient methodological details	Studies lacking a clear description of diagnostic protocols, primers, or sampling

**Table 2:** Details about country, provenience of the samples, pathogen, PCR type, and PCR primers for pathogen identification.

Year	Country	Sample	Pathogen	Diagnostic Method	Primers	References
2024	Italy	Wild boar (blood), ticks	*Anaplasma* spp.	Endpoint PCR	16SANA-F (5′-CAGAGTTTGATCCTGGCTCAGAACG-3′) 16SANA-R (5′-GAGTTTGCCGGGACTTCTTCTGTA-3′)	[77]
2023	Romania	Wild boar (blood)	*A. phagocytophilum*	Conventional PCR	LA6 (5′-GAG AGA TGC TTA TGG TAA GAC-3′) LA1 (5′-CGT TCA GCC ATC ATT GTG AC-3′)	[78]
2023	Romania	Ticks	*A. phagocytophilum*	Conventional PCR	Ge3a (5′-CACATGCAAGTCGAACGGATTATTC-3′) Ge10 (5′-TTCCGTTAAGAAGGATCTAATCTCC-3′)	[79]
			*Babesia* spp.		BJ1 (5′-GTCTTGTAATTGGAATGATGG-3′) BN2 (5′-TAGTTTATGGTTAGGACTACG-3′)	
2022	Hungary	Ticks	*Anaplasma* spp.	Real-time PCR	EphplGroEL(569)F (5′-ATGGTATGCAGTTTGATCGC-3′) EphGroEL(1142)R (5′-TTGAGTACAGCAACACCACCGGAA-3′)	[80]
			*Babesia* spp.	Conventional PCR	BJ1 (5′-GTCTTGTAATTGGAATGATGG-3′) BN2 (5′-TAGTTTATGGTTAGGACTACG-3′)	
2022	Spain	Ticks	*Anaplasma* spp.	Real-time PCR	ApMSP2-FN1 (5′-AAGGCAGTGTTGGKTAYGGTATT-3′) ApMSP2-R (5′-TTGGTCTTGAAGCGCTCGTA-3′)	[81]
			*Babesia* spp.	Conventional PCR	BJ1 (5′-GTCTTGTAATTGGAATGATGG-3′) BN2 (5′-TAGTTTATGGTTAGGACTACG-3′)	
2022	United States of America	Wild boar (blood)	*Anaplasma platys*	Conventional PCR	PLATYS-F (5′-TTGATTTTTGTCGTAGCTTGCT-3′) PLATYS-R (5′-TTGATTTCTCTCATTCCCCGT-3′)	[82]
		Ticks	*A. phagocytophilum*		ApMSP2f (5′-ATG GAA GGT AGT GTT GGT TAT GGT ATT-3′) ApMSP2r (5′-TTG GTC TTG AAG CGC TCG TA-3′)	
		Wild boar (blood)	*Babesia canis vogeli*		BTF1 (5′-TTGGCAAGGAATTAAAACTCCTTTG-3′) BTR2 (5′-CTAAGAATTTCACCTCTGACAGT-3′)	
2022	Spain	Wild boar (spleen) Ticks	*Anaplasma* spp.	Conventional PCR	16S8FE (5′-GGAATTCAGAGTTGGATC(A/C)TGG(C/T)TCAG) BGA1B-new (5′-Biotin-CGGGATCCCGAGTTTGCCGGGACTT(C/T)TTCT	[83]
			*Babesia* spp.		RLB-F2 (5′-GACACAGGGAGGTAGTGACAAG) RLB-R2 (5′-Biotin-CTAAGAATTTCACCTCTGACAGT	
2022	Brazil	Wild boar (blood) Ticks	*Anaplasma* spp.	Conventional PCR	EHR16SD (5′-GGTACCYACAGAAGAAGTCC-3′) EHR16SR (5′-TAGCACTCATCGTTTACAGC-3′)	[84]
2021	Czech Republic	Wild boar (blood) Ticks	*Anaplasma* spp.	Nested PCR	Ap-groEL-F (M13F-GAIAIIACTGAYGGTATGCAGTTTG) Ap-groEL-R (M13R-CYAIMCIYTCYYTMAGYTTTTCCTT)	[85]
2021	Sweden	Ticks	*A. phagocytophilum*	Real-time PCR	ApMSP2f (5′-ATG GAA GGT AGT GTT GGT TAT GGT ATT-3′) ApMSP2r (5′-TTG GTC TTG AAG CGC TCG TA-3′)	[86]
2021	Poland	Ticks	*A. phagocytophilum*	Conventional PCR	EHR521 (5′-TGTAGGCGGTTCGGTAAGTTAAG-3′) EHR747 (5′-GCATCCTCATCCTTTACAGCGTG-3′)	[87]
2021	Poland	Wild boar (spleen, blood), ticks (*I. ricinus*)	*A. phagocytophilum*	Conventional PCR	ApMSP2f (5′-ATG GAA GGT AGT GTT GGT TAT GGT ATT-3′) ApMSP2r (5′-TTG GTC TTG AAG CGC TCG TA-3′)	[88]
				Nested PCR	HS43 5′–ATWGCWAARGAAGCATAGTC–3′ HS45 5′–ACTTCACGYYTCATAGAC–3′	
2021	Italy	Wild boar (blood) Ticks	*A. phagocytophilum*	Real-time PCR	ApMSP2f (5′-ATGGAAGGTAGTGTTGGTTATGGTATT-3′) ApMSP2r (5′-TTGGTCTTGAAGCGCTCGTA-3′)	[89]
				Conventional PCR	groEL643f (5′-ACTGATGGTATGCARTTTGAYCG-3′) groEL1236r (5′-TCTTTRCGTTCYTTMACYTCAACTTC-3′)	
2021	Japan	Wild boar (liver, blood)	*Babesia* spp	Conventional PCR	BJ1 (5′-GTCTTGTAATTGGAATGATGG-3′) BN2 (5′-TAGTTTATGGTTAGGACTACG-3′)	[90]
2020	Turkey	Ticks	*Babesia* spp.	Conventional PCR	BJ1 (5′-GTC TTG TAA TTG GAA TGA TGG-3′) BN2 (5′-TAG TTT ATG GTT AGG ACT ACG-3′)	[91]
			*Anaplasma* spp.		MAP4AP5 (5′-ATGAATTACA GAGAATTGCTTGTAGG-3′) MSP4AP3 (5′-TTAAT TGAAAGCAAATCTTGCTCCTATG-3′)	
2018	Portugal	Wild boar (blood)	*Babesia* spp.	Conventional PCR	5′-AATACCCAATCCTGACACAGGG-3′ 5′-TTAAATACGAATGCCCCCAAC-3′	[92]
			*Anaplasma* spp.		5′-ACTGATGGTATGCARTTTGAYCG-3′ 5′-TCTTTRCGTTCYTTMACYTCAACTTC-3′	
2018	Slovakia	Wild boar (tissue) Ticks	*Babesia* spp.	Conventional PCR	BJ1 (5′-GTCTTGTAATTGGAATGATGG-3′) BN2 (5′-TAGTTTATGGTTAGGACTACG-3′)	[93]
			*Anaplasma* spp.	Real-time PCR	ApMSP2-FN1 (5′-AAGGCAGTGTTGGKTAYGGTATT-3′) ApMSP2-R (5′-TTGGTCTTGAAGCGCTCGTA-3′)	
				Nested PCR	Ge3a (5′-CACATGCAAGTCGAACGGATTATTC-3′) Ge10 (5′-TTCCGTTAAGAAGGATCTAATCTCC-3′)	
2018	Hungary	Wild boar (blood)	*A. phagocytophilum*	Real-time PCR	ApMSP2f (5′-ATG GAA GGT AGT GTT GGT TAT GGT ATT-3′) ApMSP2r (5′-TTG GTC TTG AAG CGC TCG TA-3′)	[94]
2017	Czech Republic	Ticks	*Anaplasma* spp.	Conventional PCR	M13F-GAIAIIACTGAYGGTATGCAGTTTG M13R-CYAIMCIYTCYYTMAGYTTTTCCTT	[95]
			*Babesia* spp.		TGCGCAAATTACCCAATCCTGACAC TCCAGACTTGCCCTCCAATTGGTA	
2016	Slovakia	Wild boar (blood)	*A. phagocytophilum*	Real-time PCR	ApMSP2f (5-ATGGAAGGTAGTGTTGGTTATGGTATT-3′) ApMSP2r (5′-TTGGTCTTGAAGCGCTCGTA-3′	[96]
2014	Romania	Wild boar (spleen, liver, kidney)	*A. phagocytophilum*	Nested PCR	ge9f (5′-AACGGATTATTCTTTATAGCTTGCT-3′) ge2 (5′-GGCAGTATTAAAAGCAGCTCCAGG-3′)	[97]
2014	Germany	Wild boar (blood) Ticks (*I. ricinus*)	*A. phagocytophilum*	Conventional PCR	Ge3a (5′-CACATGCAAGTCGAACGGATTATTC-3′) Ge10 (5′-TTCCGTTAAGAAGGATCTAATCTCC-3′)	[98]
			*Babesia* spp.		BJ1 (5′-GTCTTGTAATTGGAATGATGG-3′) BN2 (5′-TAGTTTATGGTTAGGACTACG-3′)	
2011	Belgium	Wild boar (spleen)	*Anaplasma* spp.	Nested PCR	EC9 (5′-TACCTTGTTACGACTT-3′) EC12A (5′-TGATCCTGGCTCAGAACGAACG-3′)	[99]
